# Prevention care management increases cancer screening among women: from efficacy to effectiveness to dissemination, implementation & scale-up

**DOI:** 10.1186/1748-5908-10-S1-A8

**Published:** 2015-08-14

**Authors:** Jonathan N Tobin, Michael L Beach, Andrea Cassells, Christina M Robinson, Xiaonan Xue, Elisa S Weiss, Allen J Dietrich

**Affiliations:** Clinical Directors Network, New York, NY 10018 USA; Geisel School of Medicine at Dartmouth, Hanover, NH 03755 USA; Albert Einstein College of Medicine, Bronx, NY 10461 USA

## Introduction

Colorectal cancer (CRC) is the second leading cause of preventable cancer-related deaths, and Blacks and Latinos are disproportionately affected. While CRC screening reduces cancer disparities, barriers exist at the system, clinician and patient levels. Prevention Care Management (PCM) has increased CRC screening rates in eleven CHCs and four Medicaid Managed Care Organizations (MMCOs).

## Objective

Three consecutive randomized controlled trials (RCTs) tested whether PCM improves CRC screening among low-income, uninsured and Medicaid-insured women aged 50-64 in Community Health Centers (CHCs) and other primary care practices and Health Plans. We present here the spectrum of research from efficacy (PCM1), to effectiveness (PCMT), to dissemination and implementation (PCM2), to scale-up and sustainability (PCM3), and now examine the contribution of patient and community-based organization (CBO) stakeholder engagement to scale-up and sustainability.

## Methods

Health education/outreach staff, initially from one practice-based research network (PBRN) and four MMCOs, and now a second PBRN, three CHCs and two CBOs, provide structured telephone support in English, Spanish or Russian, to identify barriers and facilitators to receiving CRC screening. Women receive educational materials and were followed up using chart/EHR (PCM1/PCM3) and administrative/claims (PCMT/PCM2) data.

## Results

PCM addressed barriers at patient (competing priorities, concern about the test, lack of understanding about being asymptomatic); clinician (lack of clinician recommendation); and systems (difficulty making appointments and long waiting times for procedure) levels. CRC screening increased significantly (P < 0.05) in CHCs (OR = 1.60) and Health Plans (OR = 1.44), as compared to Usual Care with no significant heterogeneity (I2 = 54.9%, p = 0.11). Overall, PCM1/PCM2 effects are stronger for Spanish-speaking women (OR = 1.92/1.81) versus English-speaking women (OR = 1.38/1.13).

Implementation Science Conclusions: PCM is robust and transferable, increases CRC screening, and can be disseminated and implemented successfully across a wide range of settings in underserved communities. Effective and sustainable PCM interventions address multi-level barriers, and should be integrated into primary care.

## Funding

New York Prevention Care Management Project (PCM1) NCI 3R01 CA87776, Dartmouth Medical School (09/01/2000-08/31/2004) New York Prevention Care Manager Translation Pilot (PCMT) NCI 3R01 CA87776-04-S1, Dartmouth Medical School (9/01/2004 -8/31/2005) New York Prevention Care Management Project 2 (PCM2) NCI R01 CA119014-01, Dartmouth Medical School (08/01/2006-07/01/2011) New York Prevention Care Management Project 3 (PCM3)/ "Collaborative Care to Reduce Depression and Increase Cancer Screening Among Low-Income Women" PCORI IH-12-11-4522, Albert Einstein College of Medicine/Montefiore Medical Center (08/01/2006-07/01/2011).

